# A Possible Role for the Asymmetric C-Terminal Domain Dimer of Rous Sarcoma Virus Integrase in Viral DNA Binding

**DOI:** 10.1371/journal.pone.0056892

**Published:** 2013-02-22

**Authors:** Ke Shi, Krishan K. Pandey, Sibes Bera, Ajaykumar C. Vora, Duane P. Grandgenett, Hideki Aihara

**Affiliations:** 1 Department of Biochemistry, Molecular Biology and Biophysics, University of Minnesota, Minneapolis, Minnesota, United States of America; 2 Institute for Molecular Virology, Saint Louis University Health Sciences Center, St. Louis, Missouri, United States of America; University of Kansas Medical Center, United States of America

## Abstract

Integration of the retrovirus linear DNA genome into the host chromosome is an essential step in the viral replication cycle, and is catalyzed by the viral integrase (IN). Evidence suggests that IN functions as a dimer that cleaves a dinucleotide from the 3′ DNA blunt ends while a dimer of dimers (tetramer) promotes concerted integration of the two processed ends into opposite strands of a target DNA. However, it remains unclear why a dimer rather than a monomer of IN is required for the insertion of each recessed DNA end. To help address this question, we have analyzed crystal structures of the Rous sarcoma virus (RSV) IN mutants complete with all three structural domains as well as its two-domain fragment in a new crystal form at an improved resolution. Combined with earlier structural studies, our results suggest that the RSV IN dimer consists of highly flexible N-terminal domains and a rigid entity formed by the catalytic and C-terminal domains stabilized by the well-conserved catalytic domain dimerization interaction. Biochemical and mutational analyses confirm earlier observations that the catalytic and the C-terminal domains of an RSV IN dimer efficiently integrates one viral DNA end into target DNA. We also show that the asymmetric dimeric interaction between the two C-terminal domains is important for viral DNA binding and subsequent catalysis, including concerted integration. We propose that the asymmetric C-terminal domain dimer serves as a viral DNA binding surface for RSV IN.

## Introduction

Retroviruses, including human immunodeficiency virus (HIV) that causes AIDS, possess an RNA genome that is reverse transcribed into viral DNA upon entering the infected host cell. The following permanent integration of this viral DNA by the viral-encoded integrase (IN) into the host chromosome is a necessary step in virus replication. In most retrovirus systems, IN first removes a dinucleotide from both termini of the linear blunt-ended viral DNA (∼10 kb), termed 3′ OH processing. The 3′ OH recessed ends expose the highly conserved CA dinucleotide on this cleaved strand. Next, the same active sites catalyze the concerted transesterification reactions of the nascent 3′ OH groups into the cell DNA. During this concerted integration event, each retrovirus system exhibits a characteristic spacing between the targeted phosphodiester bonds on opposing DNA strands, *e.g*., 6 base-pairs (bp) for avian Rous sarcoma virus (RSV), 5 bp for HIV, and 4 bp for murine leukemia virus and prototype foamy virus (PFV) [1], [2]. The different spacing likely reflects structural variation in the IN-DNA complexes that juxtapose the two viral DNA ends for concerted integration.

Most retrovirus IN proteins contain three conserved structural domains. RSV IN consists of the N-terminal domain (NTD) (residues 1–44), the catalytic core domain (CCD) (residues 50–214), and the C-terminal domain (CTD) (residues 222–286), similar in size to the corresponding domains of HIV IN. The CCD is homodimeric in all crystal structures of IN published to date, where the catalytic sites on each monomer are positioned on opposite surfaces of the dimer separated by a distance incompatible with concerted integration in most cases (for review see [3]). Biochemical and structural studies of IN from HIV and other retroviruses have suggested that a dimer of IN is responsible for integration of each viral DNA end [2], and therefore concerted integration of both viral DNA ends would require an IN tetramer [4], [5], [6]. The recent groundbreaking crystallographic work on the PFV IN-DNA complexes indeed showed a tetramer of IN bound to two viral DNA termini capable of concerted integration [7], [8]. However, despite addressing numerous fundamental questions concerning mechanisms of the retroviral IN-catalyzed reactions, the PFV IN-DNA complex structures leave an important question unanswered; why is a dimer of IN necessary for integration of each viral DNA end?

In the PFV IN-DNA complexes, all viral and target DNA interactions as well as all protein-protein contacts mediating dimerization of IN dimers to form a tetramer are made by a particular pair of IN molecules dubbed the “inner subunits” [7]. The other pair of IN molecules, the “outer subunits”, are disordered except for their CCDs that are bound to the outside faces of the inner IN subunits-DNA complex via the conserved CCD dimerization interface. As such, the series of PFV IN-DNA structures give an impression that, while the CCD of the outer subunits may be required for structural integrity, the other three domains of the outer IN subunits are dispensable [7]. SAXS analysis of the PFV intasome also suggested that the domains of the outer subunits unresolved in the crystal structure do not interact with the viral or target DNA [9]. Conversely, biochemical complementation analyses of dimeric HIV IN mutants demonstrated that not only the CCD of IN but also other structural domains, either the NTD containing the zinc-finger or the CTD that is β-strand rich, is required for both subunits in carrying out a single-ended viral DNA integration reaction into a target DNA [10], [11]. It is possible that structural requirements of the IN dimer for integration varies somewhat among different retrovirus systems.

We have structurally studied RSV IN in order to gain insights into the organization of the three domains of IN within an IN dimer. PFV IN has proven to be an excellent surrogate system for the medically relevant but highly insoluble HIV IN [12] for structural studies [7], [13]. However, the spumaviruses including PFV are most distantly related among all retroviruses to lentiviruses including HIV. PFV IN shares only a ∼15% sequence identity with HIV IN and is ∼100 amino acids (aa) larger than HIV IN, comprising an additional NTD extension domain (48 aa) and longer inter-domain linkers. Therefore, structural features of functional IN-DNA complexes distal to the active site may not be strictly conserved between PFV IN and the smaller three-domain IN including HIV and RSV [14]. RSV IN shares ∼25% sequence identity with HIV IN and the two proteins are very similar to each other in size (286 vs. 288 aa, respectively) and the lengths of inter-domain linkers [3]. Thus, structural information obtained with RSV IN could help improve understanding of how IN from HIV and closely related retroviruses function.

Although many crystal structures are available for single or two-domain fragments of HIV [15], [16], [17], RSV [18], [19], [20], simian immunodeficiency virus [21], and bovine immunodeficiency virus IN [22], a three-domain retroviral IN complete with all structural domains has not been characterized using x-ray crystallography [3]. In this report, we describe crystallographic analyses of a three-domain RSV IN as well as its CCD-CTD fragment in a new crystal form at a much improved resolution compared to previous studies. The crystal structures, combined with earlier structural studies and our *in vitro* functional analyses, suggest that the asymmetric interaction between the two CTDs is an essential feature of an RSV IN dimer for viral DNA binding and catalysis, whereas the highly flexible NTD is required for IN tetramerization to promote concerted integration.

## Results

### The Minimal 3-domain RSV IN

**Figure 1:**
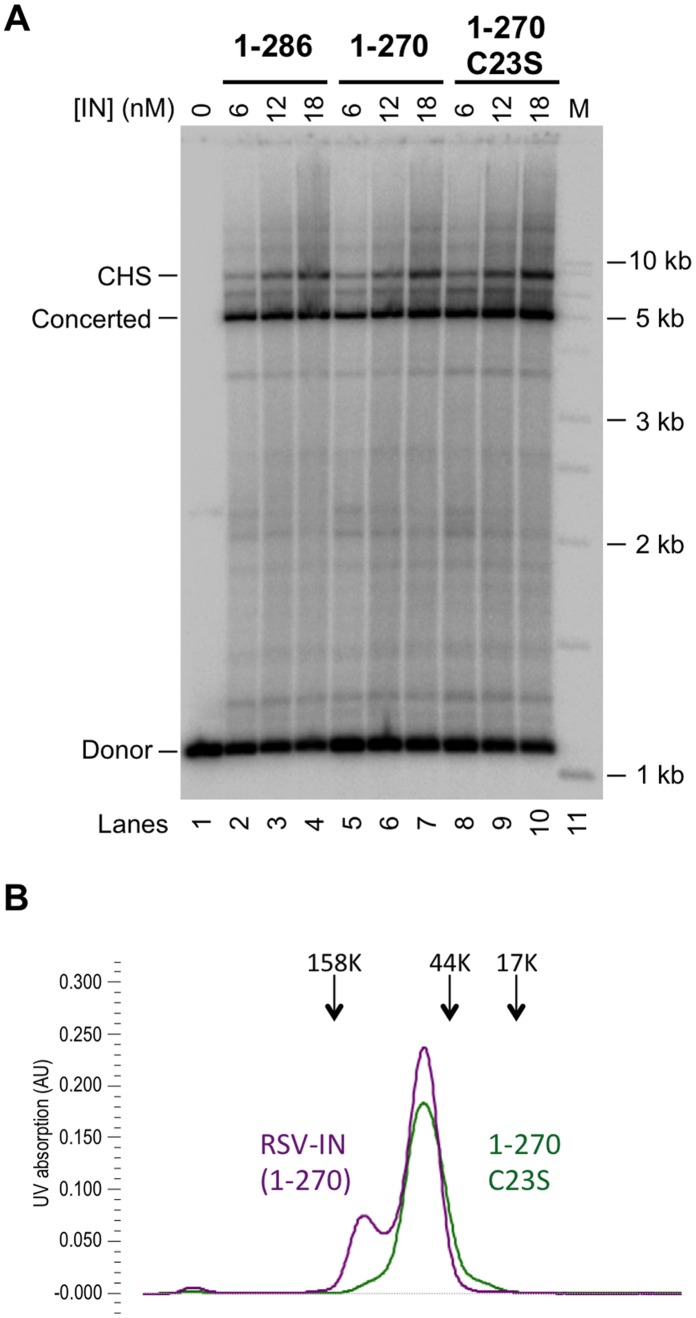
The minimal RSV IN constructs functional in concerted integration. A) *In vitro* concerted integration activities of the wild type RSV IN (1–286), the C-terminally truncated IN (1–270), and IN 1–270•C23S. The proteins were assayed for strand transfer activities using a 1.1 kb GU3 DNA substrate and with a 2.7 kb supercoiled DNA target. The concentrations (nM) of each IN are indicated at the top. The circular half-site (CHS) and concerted integration products as well as the donor substrate are indicated on the left. Lane 1 contains no IN, and in lane 11(marked M) are molecular weight markers as indicated on the right. At 18 nM IN, the percentage of donor incorporated into the concerted integration products for RSV IN 1–286, 1–270, and 1–270•C23S were 41%, 37%, and 50%, respectively. The CHS products were 10%, 9%, and 13%, respectively. The NaCl concentration in the reaction condition was 300 mM. B) Size-exclusion chromatography profiles of purified RSV IN (1–270) and IN 1–270•C23S. The proteins at 1 mg/ml were injected into a Superdex-200 column (10/300) operating with a running buffer containing 1 M NaCl. The elution positions for the molecular weight standards are indicated by arrows.

To facilitate structural characterization of RSV IN, we sought to generate a protein with less of unstructured and possibly extraneous residues. Earlier crystallographic and NMR studies showed that the extreme C-terminal region of RSV IN spanning residues 271–286, and the corresponding residues 271–288 of HIV IN, are disordered [15], [19], [23]. Thus, we generated RSV IN(1–270) lacking this flexible C-terminal “tail”. RSV IN(1–270) was overexpressed in bacteria and purified to homogeneity without using an affinity tag. An *in vitro* integration assay using a 1.1 kb viral DNA substrate and a circular target DNA showed that RSV IN(1–270) as well as its slightly more soluble point mutant RSV IN(1–270)•C23S are capable of concerted integration similarly to the full-length wild type RSV IN(1–286) (**Figure 1A**). All three proteins are also capable of inserting a single-viral DNA end into a circular target, designated circular half-site (CHS) integration. We thus concluded that the C-terminal “tail” residues 271–286 of RSV-IN are dispensable for *in vitro* integration, at least in certain reaction conditions. Analyses by size-exclusion chromatography showed that RSV IN(1–270) is in a dimer-tetramer equilibrium (**Figure 1B**), similar to the full-length wild type RSV-IN [24]. In contrast, the fully functional point mutant RSV IN(1–270)•C23S is almost exclusively dimeric, independent of protein concentration. The observations suggest that the RSV IN tetramer that forms in the absence of DNA is distinct from the IN tetramer responsible for concerted integration.

### Crystallographic Analyses of RSV IN(1–270)

**Figure 2:**
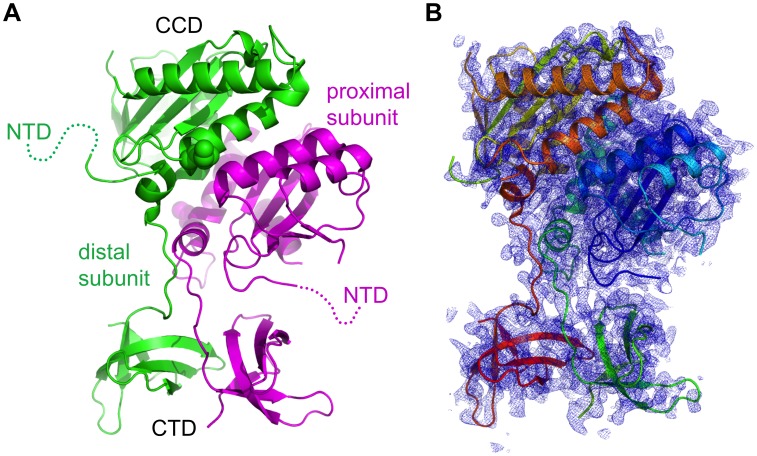
Overall structure of the RSV IN (1–270) dimer. A) Ribbon diagram showing the conformation of the RSV IN (1–270)•C23S/F199K dimer in the crystal. The NTDs are poorly ordered and thus were not modeled. The positions of K199 are indicated by spheres. B) The simulated annealing composite omit 2Fo-Fc electron density map at 2.65 Å resolution, overlaid on the ribbon model. Electron density within 1.6 Å from the protein atoms is shown, contoured at 1.0σ.

We have obtained crystals of the three-domain RSV IN(1–270) in several different conditions. Although the crystals typically grew as very thin needles not useful for x-ray diffraction experiments, the crystal morphology was improved by seeding and introducing protein mutations. Diffraction quality crystals were obtained in the presence of a solubility-enhancing F199K mutation [19]. We collected x-ray diffraction datasets on the crystals of RSV IN(1–270)•C23S/F199K and RSV IN(1–270)•L8E/C23S/F199K/W233F, and determined the structures by molecular replacement at 2.65 Å and 3.66 Å resolution, respectively, using the published domain structures of RSV/ASV IN [18], [19] (statistics for x-ray diffraction data and model refinement are summarized in **Table 1**). In the crystals, the asymmetric unit contains one RSV IN(1–270) dimer (**Figure 2**).

**Table 1:** X-ray data collection and model refinement statistics.

Structure	(1–270)•C23S/F199K	(1–270)•L8E/C23S/F199K/W233F	(49–270)E157C•DNA x-link
***Beamline***	APS 14-BM-C	APS 24-ID-C	APS 24-ID-C
***Resolution range (Å)***	50–2.65 (2.70–2.65)	50–3.66 (3.72–3.66)	50–1.86 (1.89–1.86)
***Space group***	C 2	C 2	P 2_1_ 2_1_ 2_1_
***Unit cell (Å, deg.)***	176.4, 56.2, 65.3, 90, 97.86, 90	175.5, 56.1, 65.0, 90, 97.55, 90	54.8, 67.5, 126.6, 90, 90, 90
***Total reflections***	63563	21551	158202
***Unique reflections***	18558	7023	39853
***Multiplicity***	3.4 (3.2)	3.2 (2.5)	3.9 (3.2)
***Completeness (%)***	99.37 (95.63)	95.1 (80.70)	99.21 (96.23)
***Mean I/σ(I)***	15.86 (3.19)	7.94 (3.35)	15.42 (1.76)
***Wilson B-factor***	43.79	68.78	27.96
***R_sym_***	0.08 (0.50)	0.14 (0.26)	0.06 (0.75)
***R-factor***	0.17 (0.24)	0.23 (0.31)	0.18 (0.26)
***R_free_***	0.24 (0.33)	0.26 (0.41)	0.22 (0.29)
***Number of atoms***	3402	3393	3633
***macromolecules***	3303	3393	3386
***ligands***			
***water***	99	0	247
***Protein residues***	417	435	426
***R.m.s.d. bonds (Å)***	0.008	0.008	0.007
***R.m.s.d. angles (deg.)***	1.13	1.40	1.04
***Ramachandran favored/allowed(%)***	100	96.5	100
***Ramachandran outliers (%)***	0	3.5	0
***Average B-factor***	59.10	49.30	38.90
***macromolecules***	59.60	49.30	38.50
***solvent***	44.50		44.30

Statistics for the highest-resolution shell are shown in parentheses.

The catalytic and the C-terminal domains of RSV IN(1–270) form a canted dimer very similar to that observed in the previously reported crystal structure of RSV IN(49–286) [19], despite completely different crystal packing interactions (**Figure 2A**). The two catalytic domains interact with each other through the conserved, symmetric dimerization interface observed in most crystal structures of retroviral IN reported to date [2], [15], [16], [17], [18], [19], [20], [21], [22]. In contrast, the two CTDs dimerize through an asymmetric interface and are not related by a two-fold rotational symmetry. Correspondingly, the linker segments connecting both CCDs and their CTDs adopt different conformations between the two molecules, stabilized by the “off-registered” parallel β-sheet-like interactions [19].

Whereas the final composite omit 2Fo-Fc electron density map shows clear density for the CCD and the CTD *(* **Figure 2B**), only very weak and discontinuous densities were observed for the NTD. In fact, for only one of the mutants analyzed, RSV IN(1–270)•L8E/C23S/F199K/W233F, we were able to roughly locate the NTD for one of the molecules in the RSV IN(1–270) dimer. As SDS-PAGE analyses of dissolved crystals demonstrated intact proteins without proteolysis in all cases (data not shown), the poor electron density was interpreted as a sign of flexibility of the NTDs. Due to the poor quality of the electron density map, we did not build NTDs in our models. The poorly ordered NTD appears to interact with its crystallographic symmetry-related molecule in the crystal, bridging between the two RSV IN dimers.

### Roles of the RSV IN Structural Domains

**Figure 3:**
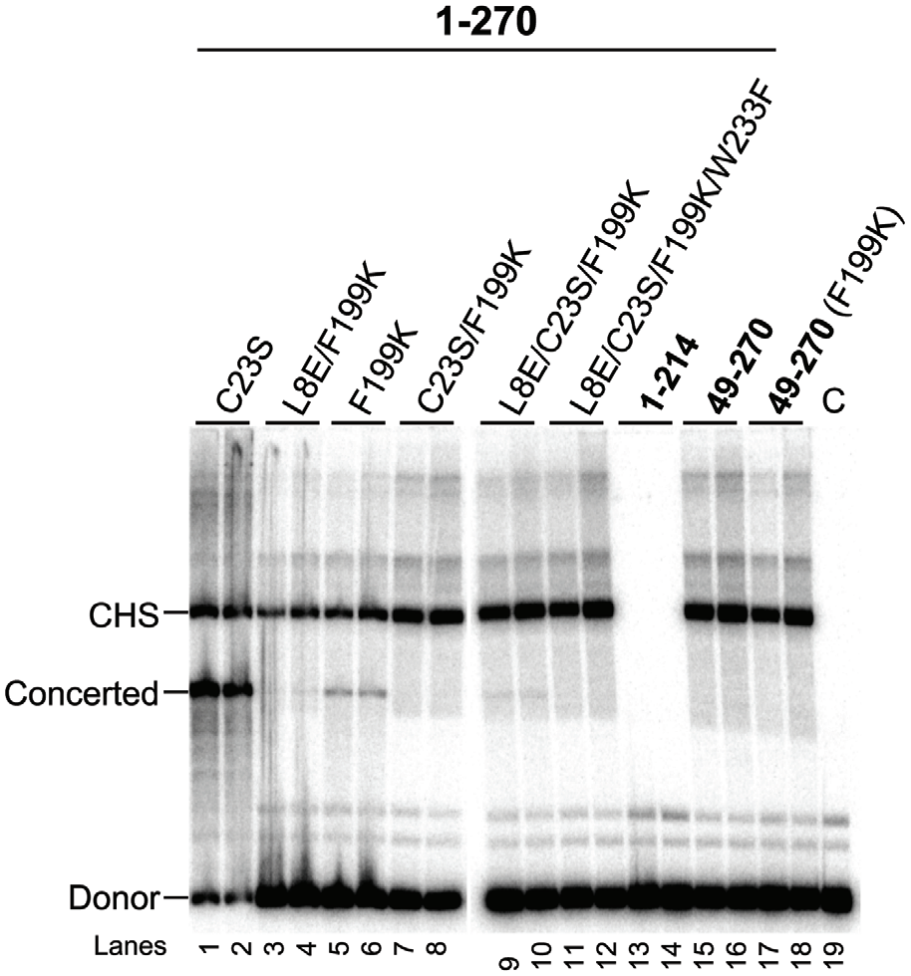
Integration activities of RSV IN (1–270) mutants and 2-domain IN constructs. RSV IN (1–270) constructs with the mutations as indicated at the top (lanes 1 to 12), and the two-domain IN construct lacking either the CTD (lanes 13 and 14) or the NTD (lanes 15 to 18) were analyzed at protein concentrations of 8 nM and 12 nM, in an assay condition containing 100 mM NaCl. The control reaction was without IN marked C (lane 19). The strand transfer products and the 3.6 kb GU3 donor are indicated on the left.

The RSV IN(1–270) constructs containing various mutations were capable of promoting the CHS integration reaction in similar fashions (**Figure 3** **, lanes 1 to 12**) (**Table 2**). To better correlate the observed structural features of RSV IN to its function, we examined the integration activities of two-domain fragments RSV IN(49–270) and RSV IN(1–214) lacking the NTD and CTD, respectively. RSV IN(1–214) was found to be completely inactive in integration reactions and produced no products under any of the conditions tested (**Figure 3** **, lanes 13 and 14**). Alternatively, RSV IN(49–270) is inactive in the concerted integration reaction but was still capable of integrating a single-viral DNA end (**Figure 3** **, lanes 15 to 18**). RSV IN with similar size NTD deletions have been shown to be capable of integrating a single-DNA end into a target substrate, although concerted integration activity was not analyzed [19], [25], [26]. Taken together, these results imply that the CCD and CTD of RSV IN are primarily responsible for viral and target DNA binding whereas the NTD is essential for IN tetramerization required for concerted integration.

**Table 2:** Catalytic activities of RSV IN constructs.

RSV IN	3′-Processing	Concerted	CHS
1–286	++++	++++	++++
1–270	++++	++++	++++
1–270 (C23S)	ND	++++	++++
1–270 (F199K)	ND	+	++++
1–270 (C23S/F199K)	ND	−	++++
1–270 (L8E/199K)	ND	−	+++
1–270 (L8E/C23S/199K)	ND	−	++++
1–270(L8E/C23S/199K/W233F)	ND	−	++++
1–270 (W213A)*	++	+	+
1–270 (P222A)	+++	+++	+++
1–270 (W242A)	++++	++++	++++
1–270 (R244A)*	++	+	+
1–270 (W259A)	−	−	−
1–270 (W259R)	−	−	−
1–270 (W259T)	−	−	−
1–270 (R263A)	+	+	+
1–270 (K266A)	+++	++	+++
1–270 (P267A)	++++	++++	++++
1–214	ND	−	−
49–270	++	−	++++
49–270(S124D/C125A/E157C/F199K)	ND	ND	ND
49–270 (F199K)	ND	−	++++
49–270 (W259A)	−	−	−

In comparison to the wild type RSV IN 1–286, strand transfer activities were reported using 300 mM NaCl and for 3′ OH processing using 100 mM NaCl.

* 1–270 (W213A) and 1–270 (R244A) had strand transfer activities comparable to IN 1–270 when assayed at 100 mM NaCl.

In the 3′ OH processing assays, MgCl_2_ was used in all assays for comparison except that MnCl_2_ was used for 49–270. ND, not determined.

Curiously, the solubility-enhancing F199K mutation used to facilitate the crystallographic analyses selectively affected concerted integration rather than CHS integration (**Figure 3** **, lanes 5 and 6**); the same apparent effect as the NTD deletion. A possible explanation would be that the residue F199 interacts with the NTD of RSV IN to mediate IN tetramerization during the concerted integration reaction. This idea is consistent with an earlier work proposing a critical ionic interaction of the corresponding HIV IN residue K186 with E11 in the NTD [27]. As RSV IN residue L8 aligns with E11 of HIV IN, a hydrophobic interaction between L8 and F199 might play a role in RSV IN tetramerization. However, our attempt to rescue the defect of F199K by a second mutation L8E to introduce a charge pair analogous to K186-E11 of HIV IN was not successful (**Figure 3** **, lanes 3 and 4**). The specific mechanism through which the NTD of RSV IN contributes to the concerted integration remains to be investigated by further structural analyses.

### Crystal Structure of RSV IN(49–270)

As the two domain fragment RSV IN(49–270) without the flexible NTD is capable of carrying out the single-end integration reaction (**Figure 3**), we sought to prepare a stable complex of RSV IN(49–270) with the viral DNA oligonucleotides for further characterization. To circumvent the problem of low sequence specificity in forming a uniform IN-DNA complex, we employed protein-DNA cross-linking [14], [28]. A thiol group was attached to the 3′ OH end of a pre-cleaved viral gain-of-function (G) U3 DNA substrate [29] and was cross-linked to a cysteine residue introduced in/near the active site of IN through a disulfide bond. The selection of the cross-linking sites is based on the chemical reactions IN is known to catalyze; the recessed 3′ OH end of the viral cleaved strand should be able to reach into the active site, since IN catalyzes the generation of this 3′ OH end by an endonucleolytic cleavage as well as its subsequent attack on the target DNA backbone. We found that cysteine introduced to replace an active site residue E157 cross-linked more readily with the thiol-modified DNA than cysteine introduced at other positions, including D64, S150, Q153, A154, or C125 present in the natural RSV IN sequence. The IN-DNA cross-linking reaction typically plateaued when ∼50% of input IN is cross-linked to an oligonucleotide representing the viral DNA terminus and does not proceed further even if excess of DNA is added, likely reflecting the fact that only one molecule within the IN homodimer takes the catalytic role (**Figure S1 A**).

**Figure 4:**
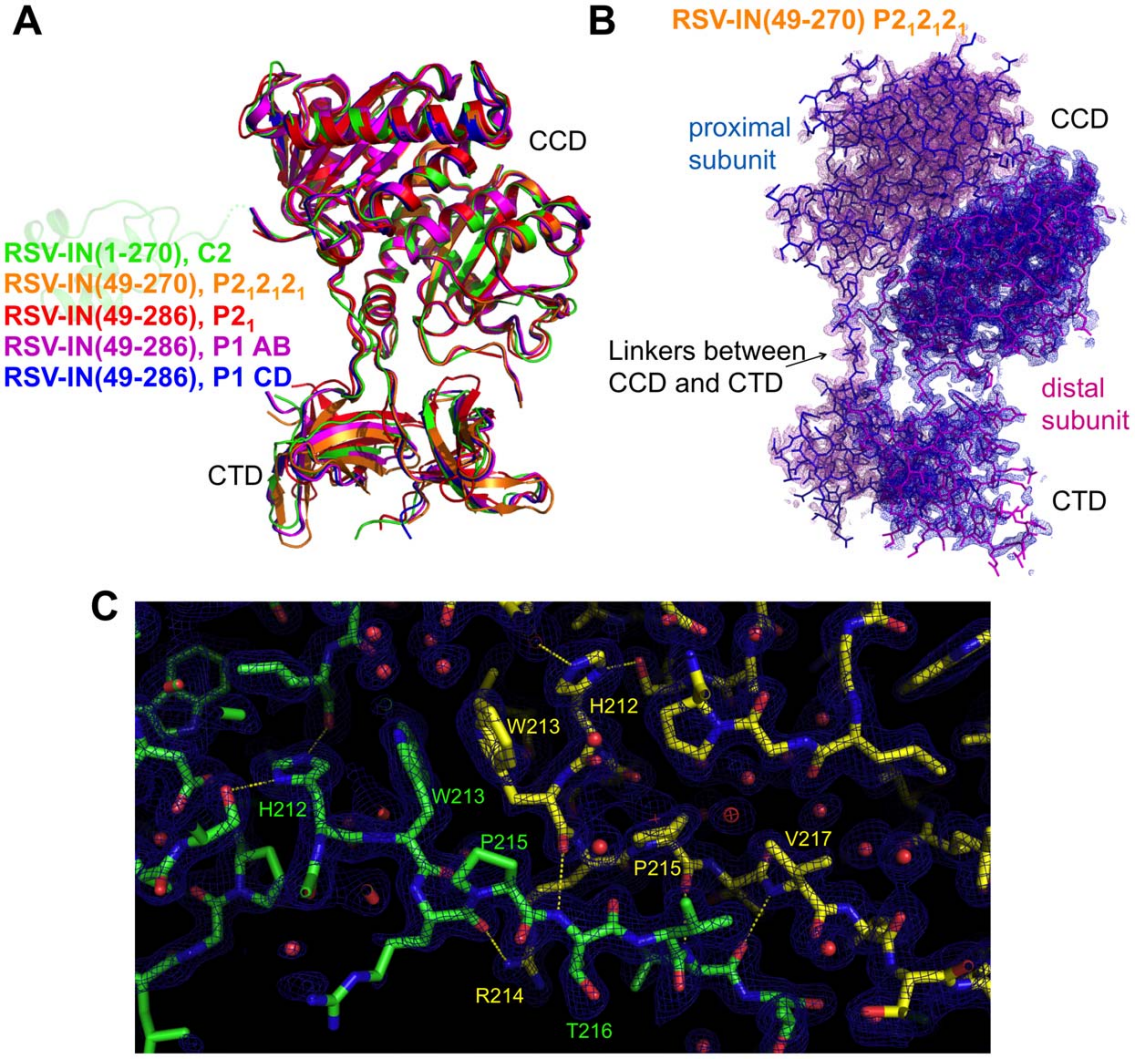
The asymmetric CCD-CTD dimer is a rigid entity. A) Superposition of various RSV IN crystal structures determined in different contexts. The construct and space group for each crystal structure is indicated, with the corresponding structures color-coded. The structures of RSV IN(49–286) were reported previously [19]. The structures of RSV IN(1–270) and RSV-IN(49–270) are from the present study. An NTD in a faded color is shown to indicate that NTD is present in the crystal of RSV IN(1–270), although poorly ordered. The relative positioning of CCD and CTD is essentially the same in all crystal structures. B) The simulated annealing composite omit 2Fo-Fc electron density map calculated at 1.86 Å resolution, overlaid on the stick model of RSV IN(49–270) dimer. Electron density within 1.9 Å from the protein atoms is shown, contoured at 0.9σ. C) A close-up view of the linkers connecting CCD and CTD in the RSV IN(49–270) dimer, with the composite omit map contoured at 1.2σ. Hydrogen-bonding interactions, as described in [19], are indicated by yellow dashed lines.

Although crystallization of a purified cross-linked IN-DNA complex has not been successful, we have been able to collect a 1.86 Å resolution dataset on a crystal of RSV IN(49–270) cross-linked *in crystallo* to a short viral DNA end substrate (5/7 hairpin DNA). The crystallized RSV IN (49–270) contained mutations S124D, C125A, E157C, and F199K. The DNA cross-linked crystals were found to be in space group P2_1_2_1_2_1_, with the mode of molecular packing distinct from that in our 3-domain RSV IN(1–270) crystal in space group C2 or the published RSV IN(49–286) crystals in either space group P1 or P2_1_ [19]. The structure was determined by molecular replacement and refined against x-ray data at 1.86 Å resolution (**Figure 4B**). From the electron density map it was evident that most IN molecules in the crystal reacted with the thiol-modified DNA. The electron density for the thiol-modified DNA is strong for a few atoms from the γ-sulfur atom of C157 but becomes progressively weak for the rest of the DNA molecule, indicating that DNA is not stably bound to the protein in a unique conformation (flexibly tethered rather than stably bound to the protein) (**Figure S1 B**). It is likely that the packing of the protein molecules in the crystal sterically interfered with productive DNA binding. Nonetheless, the structure of RSV IN(49–270) modified by DNA crosslinking in the new crystal form offers an improved resolution compared to any of the multi-domain IN crystal structures reported to date, and helps our understanding of the structure and dynamics of the RSV IN dimer as discussed below.

### Functional Significance of the CTD Dimer

When the crystal structures of different RSV IN constructs were compared, the conformations of the CCDs and CTDs were found to be very similar in all cases (**Figure 4A**). Despite different lattice contacts in the crystals, conformation of the CCDs and CTDs in our RSV IN(49–270) dimer is essentially identical to that in the RSV IN(1–270) dimer containing the C23S/F199K mutations. The backbone atoms for these two crystal structures superimpose with an r.m.s. deviation of 1.0 Å. Exactly the same conformation had also been observed in both copies of the RSV IN(49–286) dimers crystallized in space group P1, while the conformation observed in the RSV IN(49–286) dimers crystallized in a different P2_1_ form is very similar with a small tilt of the CTDs with respect to the catalytic domain dimer [19]. Their RSV IN (49–286) dimer possessed only the F199K mutation.

As noted by Yang *et al*., the relative configuration between the CCDs and CTDs of RSV IN is stabilized by a large number of hydrogen bonds made by residues in or around the linker segment [19]. Given the high similarity between all the crystal structures determined in different contexts, it seems likely that the observed conformation represents the intrinsically stable native conformation of the CCDs and CTDs, rather than an arbitral conformation captured by crystal lattice contacts. In our crystallographic model of RSV IN(49–270), the average atomic B-factors refined isotropically at 1.86 Å resolution for the CCD, CTD, and the inter-domain linker are 35.6 Å^2^, 47.9 Å^2^, and 30.7 Å^2^, respectively. The smaller B-values and the well-defined electron density (**Figure 4B, C**) for the inter-domain linker segment are consistent with the idea that the RSV IN(49–270) dimer is a rigid entity with a defined relative domain configuration. Notably, a recently published SAXS study [**30**] showed that the two-domain RSV IN(49–286) dimer in solution takes the exact conformation as observed by us and previous x-ray crystallographic studies [19], although the strictly 2-fold symmetrical RSV IN(1–286) dimer proposed in the same study [30] is not consistent with the asymmetric dimer of RSV-IN observed by x-ray crystallography.

**Figure 5:**
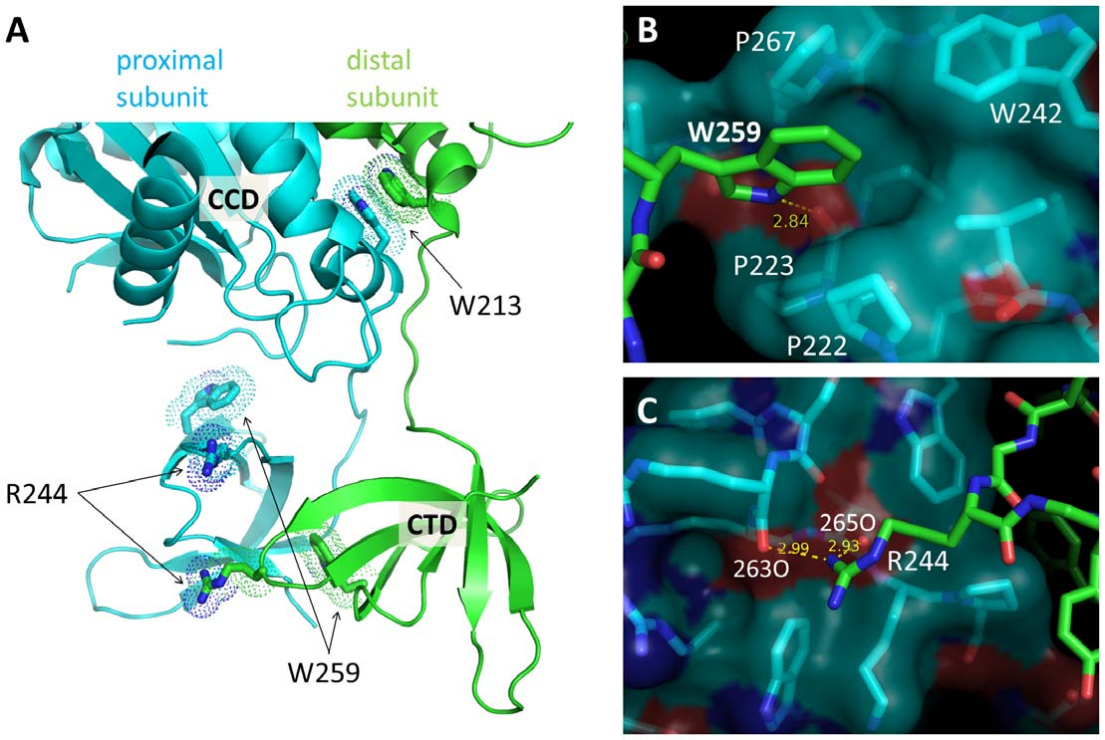
Positions of the amino acid residues around the RSV IN dimer interface mutated in this study. A) The CCD-CTD dimer of RSV IN, with W213, R244, and W259 side chains from both subunits shown in sticks. B) A close-up view of W259 and the surrounding residues P222, P223, W242, and P267 at the CTD-CTD interface. W259 is inserted into a hydrophobic pocket where it also forms a hydrogen-bond with a backbone carbonyl group of P223. C) A close-up view of the salt bridges formed by R244 from the green subunit in (A) at the CTD-CTD interface.

**Figure 6:**
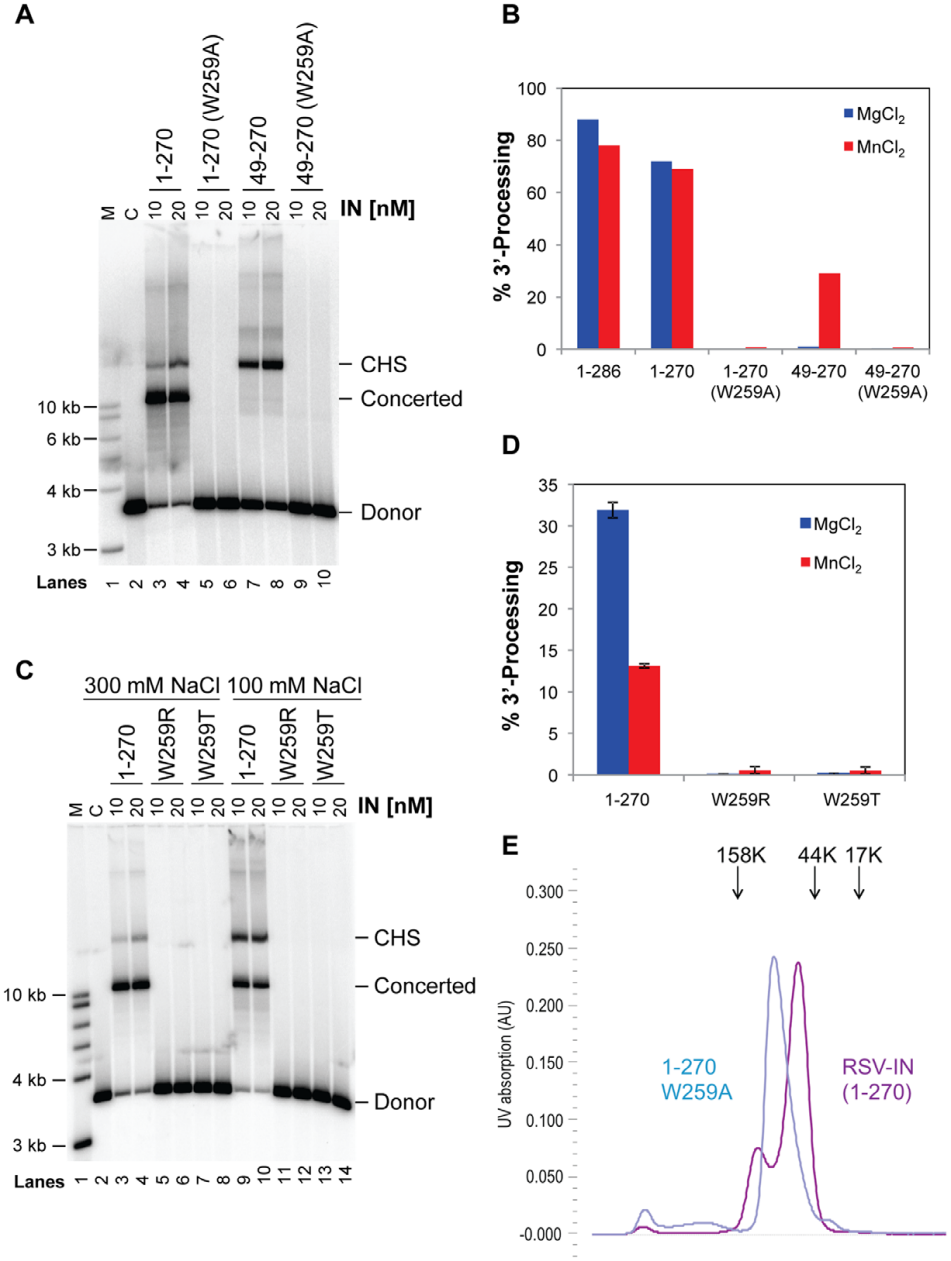
Critical role of the amino acid residue W259 in RSV IN. A) RSV IN constructs 1–270 and 49–270, and their W259A mutants, were assayed at the indicated concentrations (top) for stand transfer activities. The GU3 3.6 kb donor was used. The CHS and concerted integration products are indicated on the right. Markers are in lane 1 and the control (minus IN) is lane 2. The NaCl concentration in the reaction condition was 100 mM. B) The 3′ OH processing activity for these above constructs as well as wild type RSV IN (1–286) are shown. All of the assays contained 20 nM IN and either MgCl_2_ or MnCl_2_ as indicated. C) Integration activities of RSV IN (1–270) with W259T or W259R amino acid substitution, tested at two different NaCl concentrations. D) 3′-end processing activities of the W259T and W259R mutants, tested in the presence of either Mg^2+^ or Mn^2+^. Both mutants are completely inactive. E) Size-exclusion chromatography profile of RSV IN (1–270)•W259A, overlaid with that of RSV IN(1–270).

To assess functional significance of the observed asymmetric dimer configuration for the CCDs and CTDs, we performed mutation analyses. W259 appears to play a central role in the dimer interface between the CTDs; The tryptophan side chain inserts into the hydrophobic pocket formed by the other CTD where the Nε amide group of the indole ring makes a buried hydrogen bond with the backbone carbonyl oxygen of P223 (**Figure 5A, B**). Therefore, we introduced a W259A mutation to destabilize the dimeric interface. RSV IN(1–270)•W259A and the two domain version RSV IN(49–270)•W259A were tested in the integration assay and found to be completely inactive in both single-end and concerted integration reactions (**Figure 6A**). To distinguish whether the defect in the integration reaction is due to inability to bind viral DNA or target DNA, we further tested the 3′-end processing reaction of the W259A mutants. As both the full-length wild type RSV IN and its CCD-CTD fragment had been demonstrated to have 3′-OH processing activity in assay conditions containing Mn^++^ [19], [31], we performed the assay in the presence of either Mg^++^ or Mn^++^. In both cases, the W259A mutants showed no activity (**Figure 6B**), suggesting strongly that the mutation affected viral DNA binding and catalysis. Bojja *et. al*. recently reported similar detrimental effects of the W259A mutation of RSV IN to demonstrate a critical role of this residue in the context of a different protein-protein interaction, underscoring nonetheless the importance of W259 [30]. W259 of RSV IN aligns with T363 of PFV IN that makes van der Waals contacts with the terminal A base of the viral DNA in the PFV IN-DNA complex crystal structures [7] (**Figure S3B**). Therefore we generated two other mutants W259T and W259R to further probe potential roles of W259. We found that the effects of the W259T and W259R mutations are same as that of W259A, completely abolishing both 3′-end processing and integration activities (**Figure 6C, D**). The results suggest that W259 plays a critical role which is distinct from that played by T363 of PFV IN. Substitution of smaller (Ala, Thr) or a large highly charged (Arg) side-chain for W259 likely destabilized the C-terminal domain dimer interface to affect RSV IN functions. However, we cannot exclude the possibility that the mutations actually affected a critical function of W259 from the other molecule, which is positioned close to the N-terminus of CCD and not involved in the dimer interface (**Figure 5A**).

**Figure 7:**
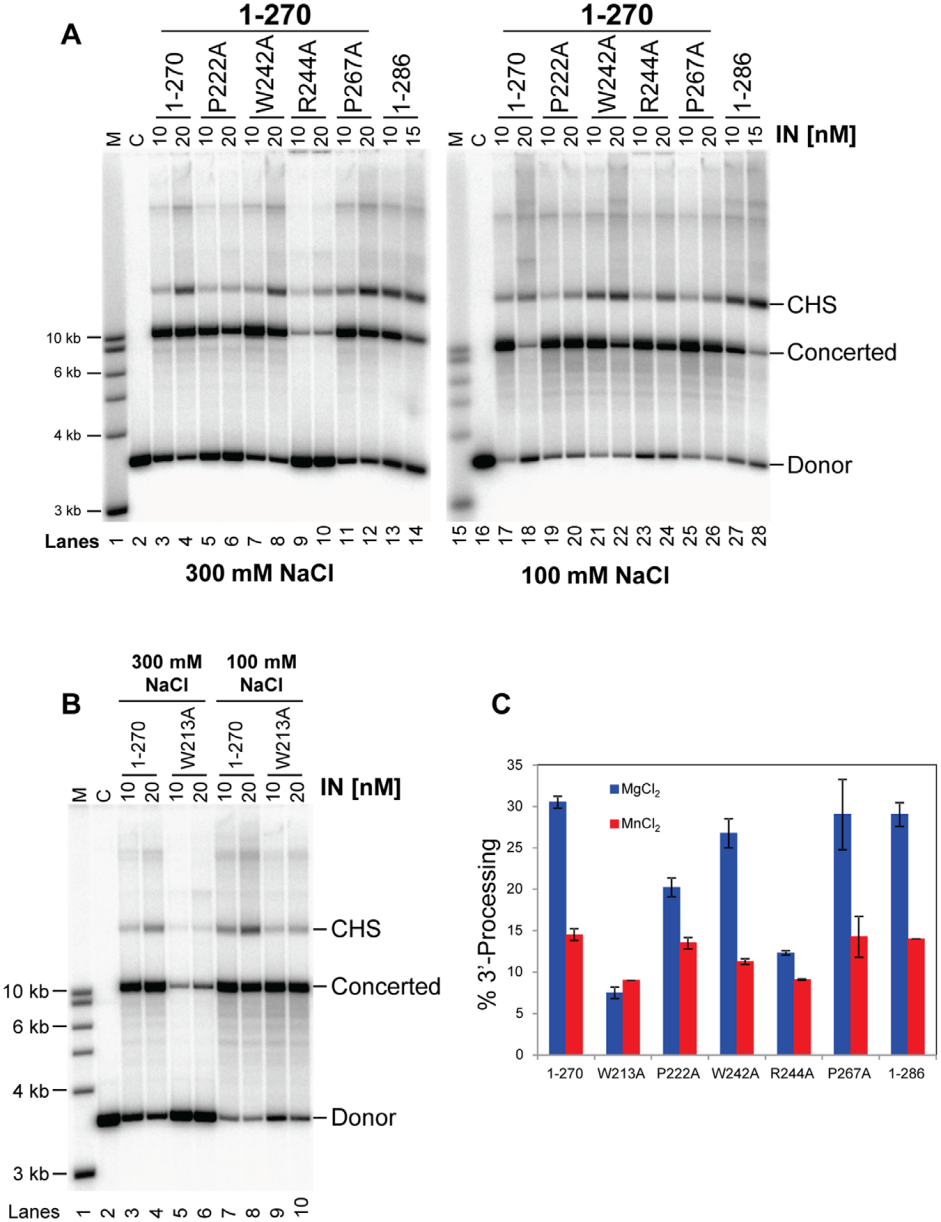
Functional analyses of RSV IN (1–270) with point mutations at residues stabilizing the CTD dimer. A) In the left panel, the RSV IN (1–270) construct without (lanes 3 and 4) or with different amino acid substitutions indicated at the top (lanes 5 to 12) were assayed for strand transfer activities at 300 mM NaCl. IN concentration was 10 nM or 20 nM and the 3.6 kb GU3 donor DNA was used. The percentage of donor incorporated into the CHS and concerted integration products at 10 nM IN was 4% and 53%, respectively (lane 3). In the right panel, the same proteins were assayed as indicated above except that the NaCl concentration was 100 mM. Lanes 1 and 15 have molecular weight markers, lanes 2 and 16 have control reactions without protein. Lanes 13, 14, 27, and 28 contain wild type RSV IN (1–286) at 10 nM or 15 nM. B) IN (1–270) and (1–270)•W213A were assayed at two different NaCl concentrations as in (A). C) The 3′ OH processing activities of IN (1–270) and all of the above mutants for the 1–270 construct were analyzed at 20 nM IN with either MgCl_2_ or MnCl_2_ at 100 mM NaCl. Wild type IN (1–286) was also assayed at 20 nM.

Using RSV IN 1–270, we also individually mutated to Ala several other residues P222, W242, and P267 that surround W259 (**Figure 5B**), R244 that forms a salt bridge with the backbone carbonyl groups of R263 and V265 across the C-terminal dimer interface (**Figure 5C**), and W213 that stack with each other to stabilize the asymmetrically associated linker segments (**Figure 4C** **,** **Figure 5A**). These mutant proteins showed various degrees of integration and 3′-end processing activities (**Figure 7A, 7B and 7C**). RSV IN(1–270)•W213A and RSV IN(1–270)•R244A showed several-fold reduction compared to RSV IN(1–270) in the 3′ OH end processing activity, and had severe defects in the integration reaction in a higher salt (300 mM NaCl) assay condition. P222A showed a slight defect in the 3′-end processing reaction. Two of the mutants, W242A and P267A, had wild type levels of activities. Size-exclusion chromatography profile showed that the W213A mutation introduced into RSV IN(1–270) causes significant reduction of apparent hydrodynamic radius (**Figure S2**) in the high-salt (1.0 M NaCl) running buffer condition we used, possibly indicating dissociation of RSV IN dimer into monomers. On the other hand, the W259A and W259T mutations rather led to an increase of apparent hydrodynamic radius (**Figures 6E and S** **2**) potentially reflecting fraying of the CTDs. The other mutations caused only subtle changes (**Figure S2**). Thus, we interpreted the results of our mutation analyses that some of the mutations destabilized, although did not necessarily completely disrupt, the asymmetric interface spanning the CTDs and the preceding linker segments, and therefore affected viral DNA binding. The milder effects of the mutations surrounding W259, as opposed to the detrimental effect of W259A itself, may reflect the relatively non-specific nature of the hydrophobic interface between the two CTDs. Of note, the W259A mutation was previously shown to cause complete dissociation of the RSV IN dimer into monomers by light scattering analysis [**30**]. It is likely that oligomeric states of the RSV IN mutants are sensitive to different solution conditions.

### Model of an RSV IN-DNA Complex

**Figure 8:**
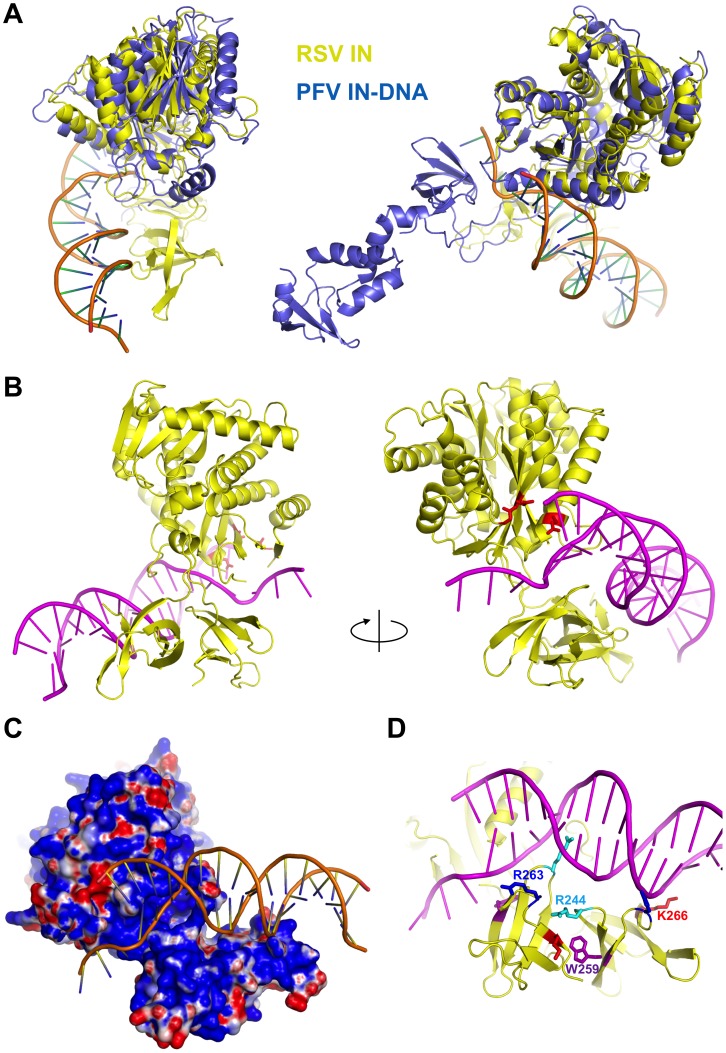
A hypothetical model of RSV IN-DNA complex. A) Superposition of the CCD dimer of RSV IN onto the CCD dimer of PFV IN in the PFV IN-viral DNA-complex [7]. RSV and PFV IN proteins are colored in yellow and slate blue, respectively, and shown in two different orientations. B) PFV IN proteins were removed from the superposition in (A), leaving the bound DNA. No adjustment was made on the position or the structure of the DNA. The catalytic residues of the proximal RSV IN subunit are shown in red sticks. C) Electrostatic surface potential (positive: blue, negative: red) is displayed for RSV IN. D) The CTD residues R244, W259, R263, and K266 that have been mutated in this study, are shown in differently colored sticks.

**Figure 9:**
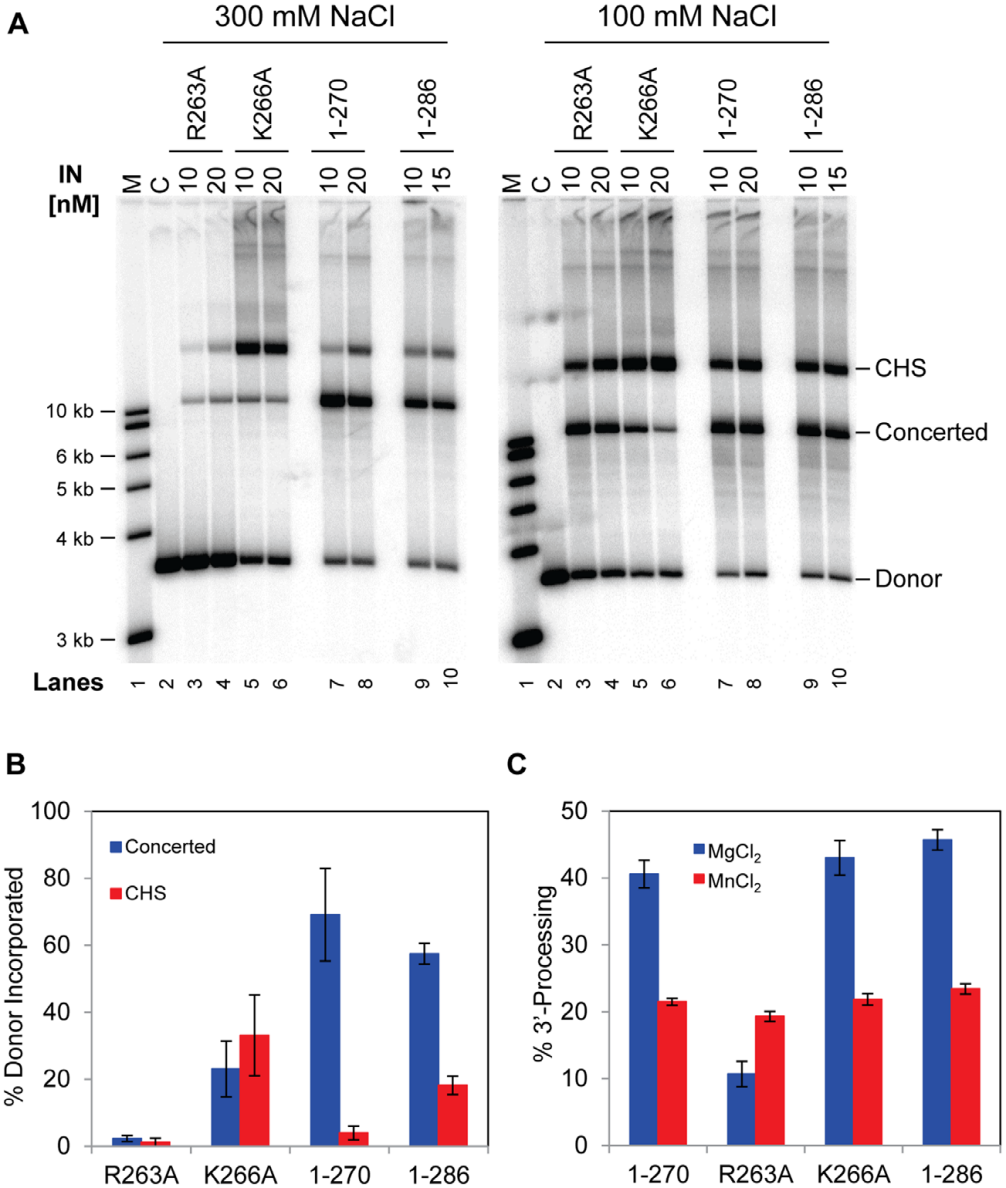
Functional analyses of RSV IN (1–270) with point mutations of basic residues on the CTD surface. A) *In vitro* integration activities of RSV IN(1–270) R263A and K266A mutants, tested at two different NaCl concentrations. B) Quantification of the assay result (300 mM NaCl) shown in (A). C) 3′-end processing activities of the R263A and K266A mutants.

Even though we do not have an experimentally determined RSV IN-DNA complex structure, the geometry in which the viral DNA terminus approaches the active site of RSV IN could be predicted based on the crystal structures of the PFV IN-DNA complex [7], as all retroviral INs catalyze essentially the same chemical reactions using the conserved active site residues. It has been demonstrated that strand transfer inhibitors directed against HIV IN potently inhibit the integration reaction by PFV IN [1], suggesting that features of viral DNA interaction close to the active site must be well conserved between retroviral INs. We thus made a hypothetical model of how the RSV IN dimer might engage a viral DNA end, by superimposing the conserved catalytic domain dimer of RSV IN onto that from the PFV IN-DNA complex then removing the PFV IN protein. The superposition places the active site residues of the proximal RSV IN subunit, the molecule in which the CCD and CTD are positioned closer to each other [19], on the corresponding residues of the “inner” PFV IN subunit accommodating the viral DNA terminus (**Figure 8A**). Curiously, the viral DNA substrate in the resulting model lies alongside the CTD dimer of RSV IN, with almost perfect shape and charge complementarity (**Figure 8B, C**). While the good fit could be purely coincidental, it seems to be consistent with our structural and mutation analyses described above that showed requirement for the stably associated CTD dimer in binding a viral DNA terminus. Furthermore, the positioning of DNA is consistent with the recently reported cross-linking data showing that R244 is located in close proximity to bases 11 or 12 on opposite strands of the viral DNA [14]. Mutations of basic residues on the dimeric CTD surface R263 and K266 (**Figure 8D**) diminish integration activities of RSV IN(1–270) particularly at a more stringent higher ionic-strength condition (300 mM NaCl), lending additional support for this hypothetical mode of viral DNA binding (**Figure 9**). Taking everything together, we would like to propose that RSV IN dimer, under some biological contexts, may bind a viral DNA end using the asymmetrically associated CTDs as a DNA-binding platform.

## Discussion

The CTD (residues 222–286) of RSV IN contains a β-strand rich region of the SH3-domain fold (222 to 270), with a “tail” region of 16 amino acids which are flexible [19]. Various deletion and single point mutations demonstrated that CTD is necessary for virus replication [32], [33], and biochemical studies have suggested its involvement in viral DNA-binding [14], [34]. Viral DNA binding to the CTD of HIV IN has also been demonstrated [35], [36], [37], [38]. Naturally occurring proteolytic processing of the “tail” of RSV or avian myeloblastosis virus IN *in vivo* up to approximately the residue E280 appears to have little or no effect on virus replication while phosphorylation of RSV IN at S282 appears to control proteolysis of IN at the very C-terminus [39]. We demonstrated here that RSV IN 1–270 possesses the same capacity *in vitro* for 3′ OH processing and concerted integration as the wild type RSV IN (1–286). The same results for these catalytic activities were obtained with truncated RSV IN (1–275) (data not shown). These above data suggest that, while CTD plays critical roles in viral DNA interaction, the entire “tail” region of RSV IN is not absolutely required for replication, similar to that shown with HIV IN [40], [41]. The identification of a minimal RSV IN construct biochemically fully active in concerted integration will facilitate further crystallographic studies, most significantly that of the RSV intasome complex including an IN tetramer and the viral and target DNA molecules.

Our structural and mutation studies collectively suggested a role for the asymmetrically associated CTD dimer of RSV IN in viral DNA binding. We showed that mutation of a basic residue R263 on the CTD surface, which is located close to the viral DNA backbone in the hypothetical viral DNA-binding model (**Figure 8D**), causes defect in 3′ OH processing and integration (**Figure 9**). Similarly, mutation of the corresponding HIV-1 IN residue R263 (R263K) has been reported to cause a slight decrease in 3′ processing and strand transfer activities [42]. The observations are consistent with direct interaction of the basic CTD surface with viral DNA. Protein-DNA cross-linking of RSV IN using either a linear or Y-shaped DNA substrate demonstrated that another CTD residue R244 primarily contacts the viral DNA at position 11 on one strand or 12 on the opposite strand, although other near DNA binding positions were also identified [30]. Mutation of R244 to Ala diminished but did not completely block 3′ OH processing or strand transfer activities (**Figure 7**) suggesting that the other DNA binding sites on the CTD do play a significant role in DNA binding. The crystal structures showed that R244 is involved in salt-bridges with the backbone carbonyl groups of R263 and V265 across the C-terminal dimer interface (**Figure 5C**), while the other R244 is free and in close proximity to the modeled DNA backbone (**Figure 8D**) because the CTD dimer is asymmetric. Thus, R244 may have dual roles to help maintain the dimer interface and make interactions with the viral DNA.

Based on different relative spatial arrangements of the CCDs and CTDs observed in the crystal structures of HIV, SIV, and RSV IN two-domain fragments [15], [19], [21], it was pointed out that these crystal structures likely show conformations impacted by crystal packing contacts [3]. We have provided data rather contrary to this idea; a particular conformation of the CCD and CTD of RSV IN is observed independently of crystal contacts, and appears to be important for viral DNA binding. The key feature of this RSV IN dimer conformation is the asymmetrically associated CTDs [19], and a modeling exercise suggested that this CTD dimer could serve as a viral DNA-binding platform (**Figure 8**). This proposed mode of viral DNA-binding is very different from how PFV IN binds the viral DNA substrate in the intasome complex [7], in which NTD plays a major role. Therefore, it remains to be further investigated whether this model actually represents how RSV IN binds the viral DNA termini in a biologically relevant context. Nonetheless, the model is consistent with the robust *in vitro* half-site integration activity observed for the two-domain fragment RSV IN(49–270) lacking NTD (**Figures 3** **,** **6**), and explains why a functional dimer of RSV IN is required for integration of a viral DNA end. Notably, RSV IN has a significantly shorter (8 aa vs. 50 aa) linker between CCD and CTD than PFV IN [3], and thus it would require unfolding of secondary structure elements in CCD or CTD to take the extended conformation observed in the PFV IN-DNA complex structure [7] (**Figure S3**). Therefore, it is conceivable that RSV IN has a somewhat different mode of viral DNA binding from PFV IN.

In the crystal structure of an HIV IN CCD-CTD fragment [15], two molecules of the CCD-CTD fragment form a Y-shaped dimer in which the two CTDs are positioned far apart from each other seemingly not making interactions. However, the CTDs from different IN dimers in fact dimerize asymmetrically (**Figure S4**) similarly to the CTD of RSV IN, in *trans* within the crystal lattice. The domain-swapped CTD dimerization observed in the HIV IN crystal may potentially reflect a generalized functional significance of the asymmetric CTD dimerization for the small three-domain retroviral INs including RSV and HIV. Further crystallographic studies, including structure determination of the IN-DNA complexes (“intasomes”) responsible for the concerted integration reaction, will be required for a more comprehensive functional understanding of IN from these retrovirus systems.

## Materials and Methods

### Protein Purification

A codon-optimized synthetic gene for the Prague A strain of RSV IN(1–270), RSV IN(49–270), or RSV IN(1–214) was inserted into the pET24a vector to generate the expression plasmids used in this study. The expression plasmids for the mutant proteins were generated by standard site-directed mutagenesis procedures. The proteins were overexpressed in *Escherichia coli* strain BL21(DE3). Transformed cells were grown in LB medium supplemented with 40 mg/L of kanamycin sulfate to an OD_600_ of ∼0.5, at which point isopropyl-β-D-thio-galactopyranoside was added to a final concentration of 1 mM to induce protein expression at an ambient temperature. The bacterial cells were collected on the next day by centrifugation, disrupted by sonication in a buffer containing 20 mM HEPES, pH7.5, 0.4 M NaCl, and 5 mM β-mercaptoethanol, then spun at 59,000×g for 1 hour. The supernatant was filtered through a surfactant-free cellulose acetate (SFCA) membrane with 0.2 µm pore-size and the filtrate was applied onto a Hi-Trap Heparin-Sepharose column. The bound proteins were eluted with a linear NaCl gradient from 0.4 to 1.5 M. The eluted RSV IN protein was concentrated by ultrafiltration, and further purified using a Superdex 200 (10/300) size-exclusion column running with 20 mM HEPES-NaOH, pH7.5, 1.0 M NaCl, 20 µM ZnCl_2_, and 5 mM β-mercaptoethanol. RSV IN(1–270) and RSV IN(49–270) were predominantly dimeric in this condition.

### Crystallography

The RSV IN crystals were produced by the hanging drop vapor diffusion method at 20°C. For the RSV IN(1–270) mutants, a concentrated (∼10 mg/mL) protein sample in the high-salt buffer was mixed with an equal volume of the well solution consisting of 20% ethanol, 100 mM imidazole-HCl, pH7.0, and 5∼10% polyethylene glycol PEG4000. Clusters of needle crystals or stacked thin plate crystals grew after 2∼3 days of incubation, during which the volume of the drop increases. Single crystals suitable for x-ray diffraction experiments were obtained by micro-seeding. For RSV IN(49–270), a protein sample (∼20 mg/mL) was mixed with the well solution consisting of 10 ∼20% ethanol, 100 mM Tris-HCl, pH8.0, and 5% PEG4000. The crystals of RSV IN (49–270) carrying a particular set of mutations S124D/C125A/E157C/F199K were transferred to a soaking/cross-linking solution containing 5% ethanol, 10% PEG4000, 100 mM Tris-HCl, pH8.5, and 0.5 mM of thiol-modified DNA in the disulfide (S-S) form. The oligonucleotides (“hairpin5/7″: 5′- AATGTTGGAACAACA-3′ or “hairpin3/5″:5′- AATGTGAAACA-3′) carrying the 3′ thiol modifier C3 S-S modification mimicked the terminal sequence of a processed viral DNA end, with the cleaved and non-cleaved strands linked by a tri-nucleotide (5′-GAA-3′) hairpin. All crystals were cryoprotected by gradually introducing glycerol into the drops to a final concentration of 20∼25%, and flash cooled in liquid nitrogen. X-ray diffraction data were collected at the beamlines 24ID-C or 14BM-C of the Advanced Photon Source (Argonne, IL), and processed using the HKL2000 suite [43]. Molecular replacement calculations were performed with PHASER [44], using the previously published crystal structures of the ASV/RSV IN CCD and CTD [18], [19] as search models. Atomic models were built using COOT [45] and refined using REFMAC5 [46]. The quality of electron density for NTD was too poor to allow model building. The final model for the RSV IN(49–270) crystal form includes residues 52–145, 154–269 (chain A), 54–269 (chain B), and that for the RSV IN(1–270) crystal form includes 52–145, 154–269 (chain A), 54–146, 154–204, 206–268 (chain B). A summary of crystallographic data and model refinement statistics is shown in Table 1. The atomic coordinates for RSV IN(1–270) and RSV IN(49–270) have been deposited in the RCSB protein data bank with the accession code 4FW2 and 4FW1, respectively. The structure figures were produced using PYMOL [47]. Electrostatic potentials were calculated using APBS [48].

### Concerted Integration Assay

The assay conditions for concerted integration using RSV IN were previously described [49], [50]. All IN concentrations used are expressed as dimers. Briefly, the assays were performed with either a linear 1.1 or 3.6 kb DNA donor substrate that possessed a single U3 LTR DNA end and was labeled with ^32^P at the 5′ end. The substrates were produced by NdeI digestion of a circular plasmid producing a 2 bp recessed U3 end. The U3 end was modified on the cleaved strand at nucleotide position 6 (T to A) producing a gain-of-function (G) mutation that possesses several-fold higher catalytic activity than the wild type U3 sequences [29] . The G U3 mutation does not affect virus replication and integration [51]. Briefly, RSV IN (10 nM to 20 nM ) and donor DNA (0.5 nM) were preassembled at 14°C for 15 min in 20 mM HEPES, pH7.5, 10 mM MgCl_2_, 5 mM DTT, 8% PEG6000, 100 or 300 mM NaCl. Both IN and NaCl concentrations affect the observed concerted integration activity. Upon addition of supercoiled target DNA (1.5 nM), strand transfer was for 30 min at 37°C. Reactions were stopped with EDTA to a final concentration of 25 mM and samples were deproteinized. Strand transfer products were separated on 1.3% agarose gel, dried, and analyzed by a Typhoon Trio Laser Scanner.

### 3′ OH Processing Assay

The assay conditions for RSV IN 3′OH processing activity was described earlier [29]. Briefly, a 4.6 kb plasmid containing the wt U5 and GU3 circle junction was digested with NdeI producing a 3′ OH recessed donor substrate. The DNA ends were filled in with [α-^32^P]TTP and cold dNTPs to make blunt ends. The 3′ OH processing activity was determined by the release of the terminal labeled dinucleotide. IN (10 nM to 20 nM) was incubated in a buffer (100 µl) containing 100 mM NaCl at 14°C for 45 min, followed by incubation at 37°C for 30 min. The reactions were stopped by adding EDTA to 25 mM and precipitated by adding 2 µl single strand DNA (2 µg) and 100 µl 20% TCA in dry-ice ethanol bath for 30 min. The DNA sample was subjected to centrifugation at 14 K for 20 min at 4°C and 100 µl supernatant as well as the pellet was measured for radioactivity. The percentage of released dinucleotide was calculated.

### Size-exclusion Chromatography

Proteins at 1 mg/mL were injected into the Superdex 200 (10/300) size-exclusion column operating at 4°C with the buffer containing 20 mM HEPES-NaOH, pH7.5, 1.0 M NaCl, 20 µM ZnCl_2_, and 5 mM β-mercaptoethanol. The following molecular weight standards were used for column calibration; bovine γ-globulin (158 K), chicken ovalbumin (44 K), and horse myoglobin (17 K).

## Supporting Information

Figure S1 **RSV IN-DNA cross-linking.** A) SDS-PAGE analysis of covalent IN-DNA complexes. RSV IN(49–270) with E157C mutation readily forms a disulfide linkage in solution with 3′-terminal thiol-modified viral DNA substrate of various lengths. The protein additionally had the following amino acid substitutions; S124D, C125A, and F199K. The DNA substrates had the GU3 viral end sequence [29], either supplied as single oligonucleotide (hp 8/10 or hp 10/12; the catalytic and non-catalytic strands are joined by a hairpin at the distal end) or two separate oligonucleotides. The gel was run in a non-reducing condition and stained with Coomassie blue. B) RSV IN(49–270) crosslinked *in crystallo* to a short viral DNA (hp 5/7) shows additional electron density on the C157 side-chain due to the cross-linked moiety. The simulated annealing composite omit 2Fo-Fc map is shown, with a few atoms built in the density beyond the γ-sulfate atom of C157 connected through a disulfide linkage.
(TIF)

Figure S2 **Oligomeric states of various RSV IN(1–270) mutants.** Size exclusion chromatography profiles of RSV IN(1–270) with and without amino acid substitutions. Profile for each mutant is overlaid with that for RSV IN(1–270). The proteins at 1 mg/ml were injected into a Superdex-200 column (10/300) operating with a running buffer containing 20 mM HEPES-NaOH, pH7.5, 1.0 M NaCl, 20 µM ZnCl_2_, and 5 mM β-mercaptoethanol. The elution positions for the following molecular weight standards are indicated by arrows; bovine γ-globulin (158 K), chicken ovalbumin (44 K), and horse myoglobin (17 K).
(TIF)

Figure S3 **Structural comparison between PFV and RSV IN.** A) Structures of RSV and PFV IN CCD and CTD, individually compared. The last residues of CCD and the first residues of CTD are labeled. B) Superposition of the CTDs. W259 of RSV IN and T363 of PFV IN are shown in sticks. C) Relative positionings of CCD and CTD. In the PFV IN-DNA complex [7], the ending residue of the last α-helix in CCD and the starting residues of the first β-strand in CTD are separate by ∼50 Å, and the intervening linker residues make viral DNA interactions. In the DNA-free RSV IN dimer structure, the CCD and CTD are positioned closer, corresponding to much fewer residues comprising the linker segment. As 8 amino acids (residues 215 to 222) are not enough to span ∼50 Å in space, for RSV IN to take the same CCD-CTD configuration as observed in PFV IN, the last α-helix of CCD needs to be unfolded.
(TIF)

Figure S4 ***Trans*** **interactions between CTDs of HIV IN.** CTD-CTD interaction observed in the crystal structure of the HIV IN 2-domain (CCD-CTD) fragment [15]. Crystallographically equivalent molecules are shown in the same color. The red oval highlights the CTD-CTD contact made *in trans* within the crystal lattice.
(TIF)
