# Supplementary figures and images for: A Possible Role for the Asymmetric C-Terminal Domain Dimer of Rous Sarcoma Virus Integrase in Viral DNA Binding
Source: PLoS One. 2013 Feb 22;8(2):e56892. doi: 10.1371/journal.pone.0056892 (PMC3579926; doi:10.1371/journal.pone.0056892)

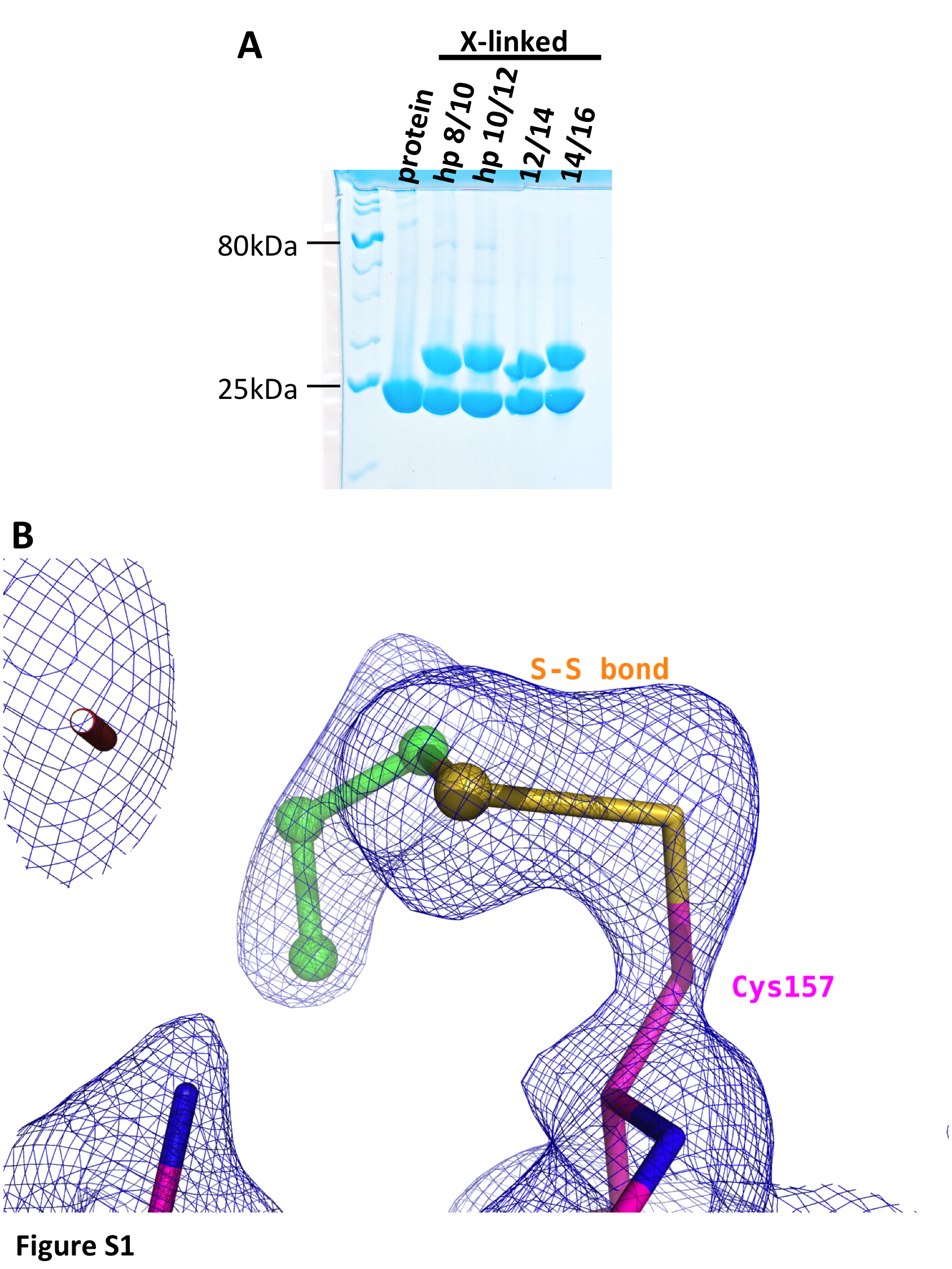

Supplement: Figure S1 — RSV IN-DNA cross-linking. A) SDS-PAGE analysis of covalent IN-DNA complexes. RSV IN(49–270) with E157C mutation readily forms a disulfide linkage in solution with 3′-terminal thiol-modified viral DNA substrate of various lengths. The protein additionally had the following amino acid substitutions; S124D, C125A, and F199K. The DNA substrates had the GU3 viral end sequence [29], either supplied as single oligonucleotide (hp 8/10 or hp 10/12; the catalytic and non-catalytic strands are joined by a hairpin at the distal end) or two separate oligonucleotides. The gel was run in a non-reducing condition and stained with Coomassie blue. B) RSV IN(49–270) crosslinked in crystallo to a short viral DNA (hp 5/7) shows additional electron density on the C157 side-chain due to the cross-linked moiety. The simulated annealing composite omit 2Fo-Fc map is shown, with a few atoms built in the density beyond the γ-sulfate atom of C157 connected through a disulfide linkage. (TIF) [file pone.0056892.s001.tif]

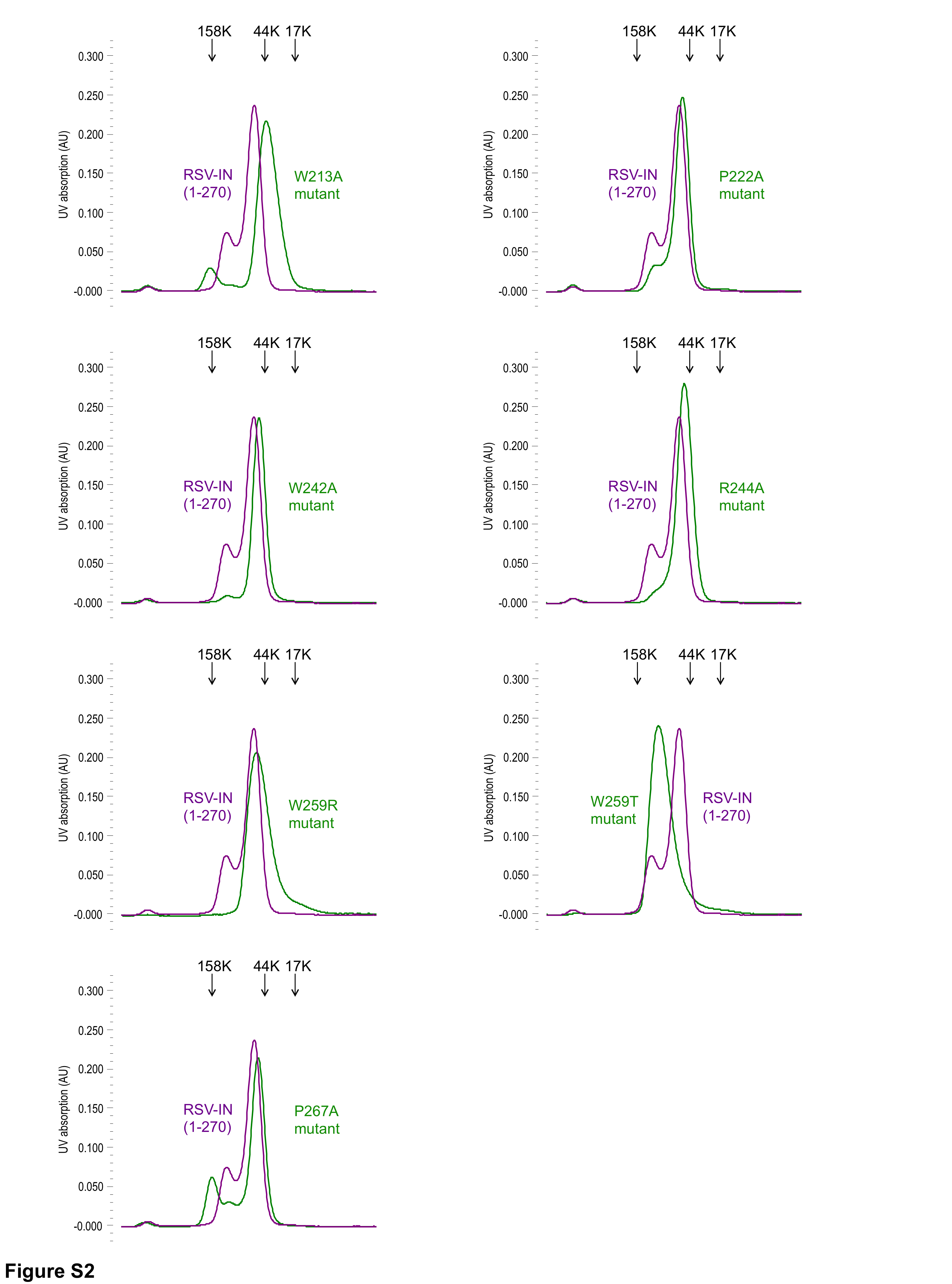

Supplement: Figure S2 — Oligomeric states of various RSV IN(1–270) mutants. Size exclusion chromatography profiles of RSV IN(1–270) with and without amino acid substitutions. Profile for each mutant is overlaid with that for RSV IN(1–270). The proteins at 1 mg/ml were injected into a Superdex-200 column (10/300) operating with a running buffer containing 20 mM HEPES-NaOH, pH7.5, 1.0 M NaCl, 20 µM ZnCl2, and 5 mM β-mercaptoethanol. The elution positions for the following molecular weight standards are indicated by arrows; bovine γ-globulin (158 K), chicken ovalbumin (44 K), and horse myoglobin (17 K). (TIF) [file pone.0056892.s002.tif]

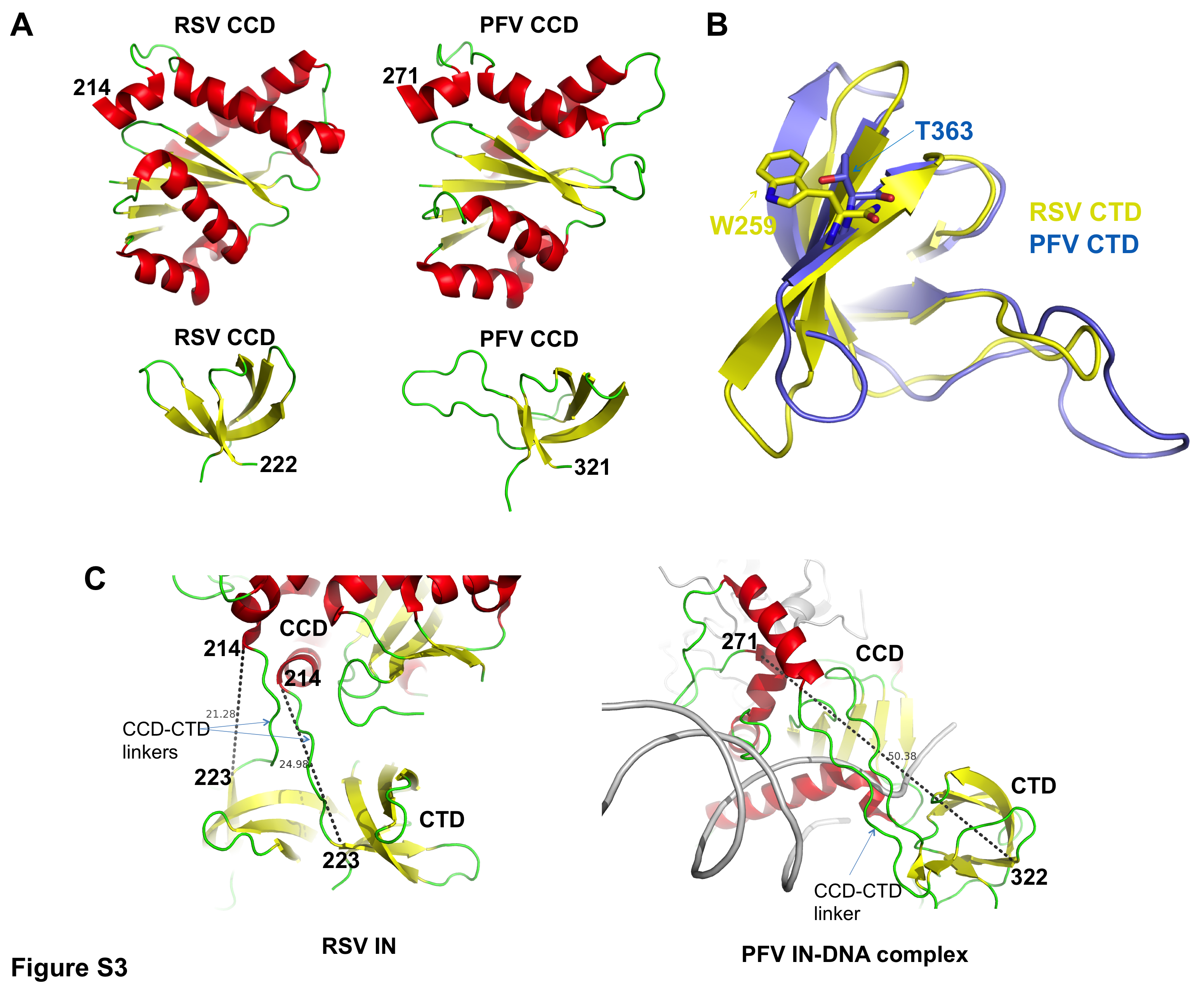

Supplement: Figure S3 — Structural comparison between PFV and RSV IN. A) Structures of RSV and PFV IN CCD and CTD, individually compared. The last residues of CCD and the first residues of CTD are labeled. B) Superposition of the CTDs. W259 of RSV IN and T363 of PFV IN are shown in sticks. C) Relative positionings of CCD and CTD. In the PFV IN-DNA complex [7], the ending residue of the last α-helix in CCD and the starting residues of the first β-strand in CTD are separate by ∼50 Å, and the intervening linker residues make viral DNA interactions. In the DNA-free RSV IN dimer structure, the CCD and CTD are positioned closer, corresponding to much fewer residues comprising the linker segment. As 8 amino acids (residues 215 to 222) are not enough to span ∼50 Å in space, for RSV IN to take the same CCD-CTD configuration as observed in PFV IN, the last α-helix of CCD needs to be unfolded. (TIF) [file pone.0056892.s003.tif]

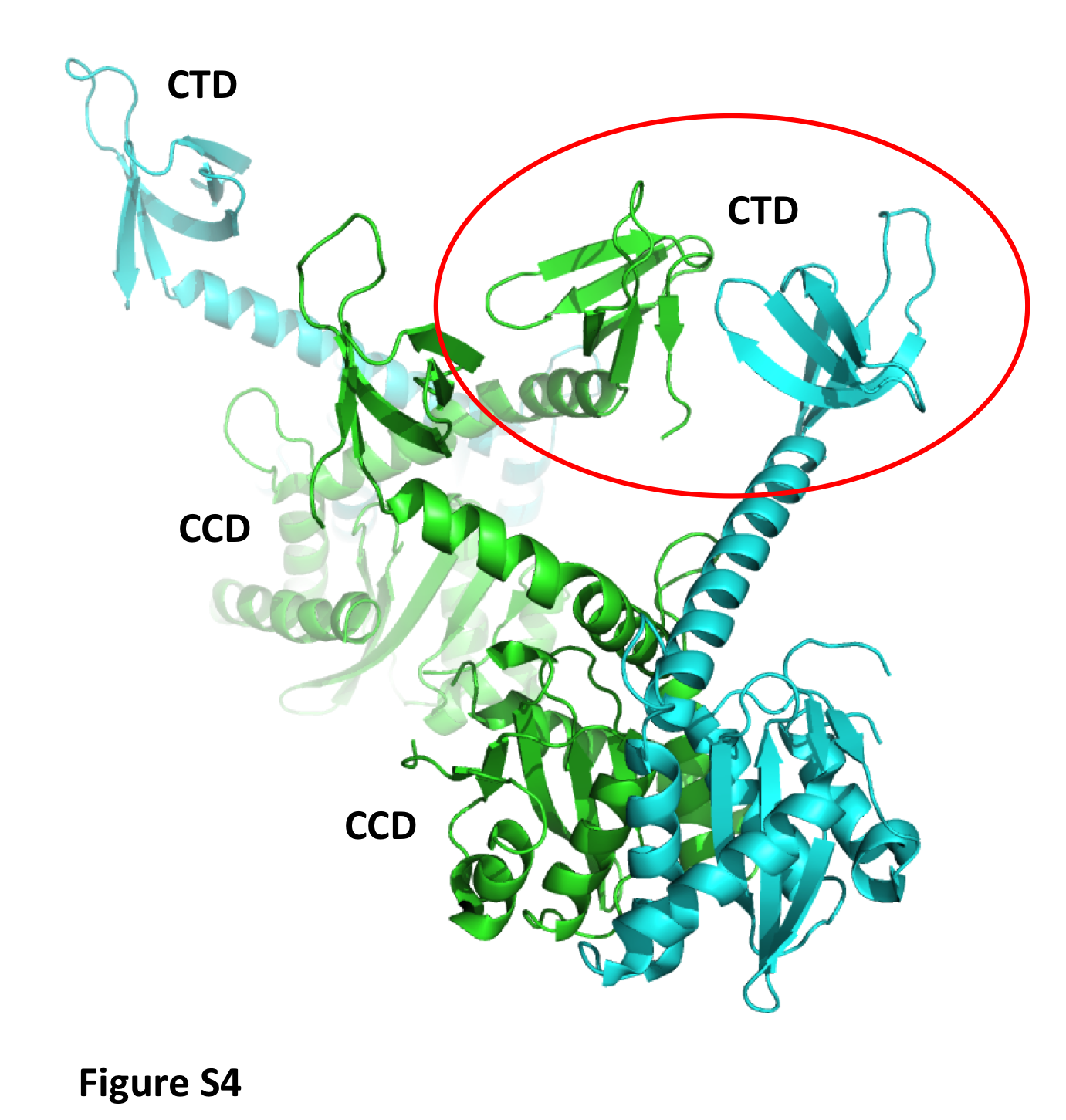

Supplement: Figure S4 — Trans interactions between CTDs of HIV IN. CTD-CTD interaction observed in the crystal structure of the HIV IN 2-domain (CCD-CTD) fragment [15]. Crystallographically equivalent molecules are shown in the same color. The red oval highlights the CTD-CTD contact made in trans within the crystal lattice. (TIF) [file pone.0056892.s004.tif]
